# Socioeconomic Status Index to Interpret Inequalities in Child Development

**Published:** 2017

**Authors:** Mahbobeh AHMADI DOULABI, Firoozeh SAJEDI, Roshanak VAMEGHI, Mohammad Ali MAZAHERI, Alireza AKBARZADEH BAGHBAN

**Affiliations:** 1 PhD Candidate of Pediatric, Neurorehabilitation Research Center, University of Social Welfare & Rehabilitation Sciences, Tehran, Iran; 2 Pediatric Department, Pediatric Neurorehabilitation Research Center, University of Social Welfare & Rehabilitation Sciences, Tehran, Iran; 3 Psychology Department, Shahid Beheshti University. Tehran, Iran; 4 PhD in Biostatistics, Proteomics Research Center, Department of Basic Sciences,School of Rehabilitation Sciences,Shahid Beheshti University of Medical Sciences,Tehran, Iran

## Abstract

**Objective:**

There have been contradictory findings on the relationship between Socioeconomic Status (SES) and child development although SES is associated with child development outcomes. The present study intended to define the relationship between SES and child development in Tehran kindergartens, Iran.

**Materials & Methods:**

This cross-sectional survey studied 1036 children aged 36-60 month, in different kindergartens in Tehran City, Iran, in 2014-2015.

The principal factor analysis (PFA) model was employed to construct SES indices. The constructed SES variable was employed as an independent variable in logistic regression model to evaluate its role in developmental delay as a dependent variable.

**Results:**

The relationship between SES and developmental delay was significant at P=0.003. SES proved to have a significant (P<0.05) impact on developmental delay, both as an independent variable and after controlling risk factors.

**Conclusion:**

There should be more emphasis on developmental monitoring and appropriate intervention programs for children to give them higher chance of having a more productive life.

## Introduction

Socioeconomic Status (SES), as a major risk factor, has gained the attention of politicians, managers and researchers throughout the healthcare system (1). SES has been studied from three perspectives: the initial exposure, a risk factor health outcome, and a confounding factor (2). Numerous studies have confirmed SES to be a predictor of child development. Different data support the relationship between SES and child development (3-5).

Children, the most important national focus of each society, determine the future of their society; therefore, their childhood development is an essential component to maintaining health throughout their life (6, 7).

Child development is a process in which brain and nervous system undergo integrated changes due to structural and functional complexity. Subsequently, children acquire new skills and capacity, their compatibility increases and they reach behavioral and functional maturation. Development consists of several domains defined by acquiring specific skills which child gradually learns at their appropriate age (8-12), included physical, social, emotional, cognitive and language domains (13).

The brain growth and development during the early years after birth is rapid and these years are an opportunity for the brain to grow, develop and achieve optimal development, hence, the brain becomes susceptible to negative environmental factors (6, 14, 15). Thus, any impairment in child development can affect child’s health as well as the societies at a larger scale. Investing in child development programs would reduce the cost and burden of chronic diseases and disabilities. Such investment would also improve SES of the society by turning a child into a healthy productive adult and it will compensate several times more than the cost in time (7, 14, 16).

Over 200 million under-five children might never reach full potential of self-cognitive development due to poverty, precarious health, nutrition and lack of environmental stimulation (17). Children suffering from poverty would also face risks of environmental and biological factors, which have accumulative and dynamic impacts on neuro psychomotor development (3, 18-23).

Speech disorders, learning disabilities and emotional disorders are reported in 15-18% of children in different societies. There are also severe psychosocial complications in 15% of the children (24-28) which might also rise up to 30% in at risk children. The prevalence rate of developmental delay in Iranian children ranges from 7% to 26.3% in different cities (29-33).

A meta-analysis study showed 14.6% cumulative frequency in different dimensions of developmental delay in Iranian children (34). Eight percent of children suffer from one or more developmental disorders from birth to 6 yr of age (35). 

Average percentage of children delayed in the communication, gross motor, fine motor, problemsolving and social-personal domains was 3.87%, 4.04%, 4.31%, 4.15% and 3.69%, respectively (36). 

Children, especially younger ones, are more susceptible to SES and poverty as these two factors would increase the risk of complications such as mental health disorders in children (37). In addition, poverty and low SES are reported to be associated with higher risk of mental health problems (38). Children with low SES also lack access to cognitive experiences and stimulations since having access to these cultural materials acts as a mediator between family income and children’s intellectual growth, academic achievement, as well as behavioral problems (3).

Lower family SES causes higher incidence of developmental delay in children. Low SES would increase unhealthy behaviors, inadequate nutrition, and failure to properly use health care, maternal diseases and drug abuse, and consequently, increase developmental delays (4, 39-41). Mother’s lower levels of education, low income and poor housing conditions are significantly correlated with child’s developmental delay (42). Poverty increases child’s exposure to biological and psychological risk factors, lead to developmental disorders by changing the brain structure and functioning (20). Children living below the poverty line are 1.3 times more prone to suffer from developmental delays or learning disabilities in comparison with non-poor children (43). Poverty can also deeply affect cognitive development and longterm poverty might lead to significant damage in this regard (44). Insufficient family income means lack of financial resources, besides being a stressor for parents and preventing provision of care for their child (45). Stress, anxiety and depression in parents, especially mothers, are associated with developmental disorders in children (46). Furthermore, children living in poverty are more exposed to family conflicts, violence, separation, instability and chaotic family. These children also experience less social support (47). 

Few studies have been conducted on the association between SES and child development in Iran and have considered few factors to evaluate SES (31, 32). Therefore, the present research studied SES as an independent variable on child development using the principal component analysis of data provided by studying children aged 36-60 months of age in Tehran, Iran.

## Materials & Methods

This cross-sectional descriptive study was conducted from Apr 2014 to Feb 2015 in kindergartens across Tehran, Iran.

The present research studied 1036 paired-samples (parent-child) through a multistage sampling technique.

The samples were all Iranian and children aged 36 to 60 months, living with both parents, and had no recognized developmental disorders due to genetic syndrome, etc. 

Simple random sampling technique was employed to choose 43 kindergartens out of all kindergartens in Tehran (North, South and Center), children (and their parents) who met the inclusion criteria were recruited.

The data collection instruments included parents-child demographic inventory, socioeconomic questionnaire. 

The demographic inventory included parents’ general information (age, educational attainment, job, gravidity and parity, and history of abortion). The socioeconomic status was assessed by studying income, price square feet residential ground, infra-structure, hosing, number of family, parental education, number of cars, and personal computer The reliability of the demographic questionnaire was evaluated through content validity using scientific resources and experts’ opinion. The validity and reliability of the demographic, socioeconomic status, and child specification questionnaire were determined using content validity and test-retest. (The correlation coefficient was 93-97% from 10 checklists). 

ASQ is currently the most widely used. Sensitivity of the ASQ test is 75% in high risk group and 100% in the community group, with specificity of 95% and 90%, respectively (48) .Validity of this test varies from 76% to 88%. In addition, ASQ includes 19 different questionnaires that can screen developmental status of children from 4 to 60 months in five different domains: communication, gross motor, fine motor, problem solving and personal-social skills. Each domain is evaluated by six questions on what the child can or cannot do. They are selected to be representatives of a developmental quotient of 75-100%. The answer of parents to each question is “yes” to indicate that the child does the special behavior of this item, ‘sometimes’’ to indicate an occasional or emerging response and ‘‘not yet’’ to indicate that their child does not yet do the behavior, with a respective score of 10, 5 or 0 points. 

Then, scores of each item summed and final score in each domain is compared to cut-off points of the ASQ guidelines. The score on any domain below the cut-off point or higher than two standard deviations below the mean of the reference group, is considered abnormal and referral for further evaluation (49-53).

ASQ is a reliable tool with Cronbach’s alpha of 0.86 and reliability of 0.93 for Iranian children (54). The reliability of this scale in present study was obtained as 0.88, using the test-retest method. 

The researcher got required permissions to conduct the study and obtained written informed consents from kindergarten teachers after introducing the objectives and asking for their cooperation. The mother/child Demographic Questionnaire, Socioeconomic Survey Questionnaire, and the age-appropriate Ages and Stages Questionnaire (ASQ) were given to mothers to be filled out at home (within four days). The researcher calculated the scores in ASQ based on age-appropriate cut off values. Mothers were informed of the results and they were referred to responsible organizations if the score was below the cutoff value.

The principal component analysis (PCA) was used to create SES variable. PCA is a multivariate statistical method widely used during recent years to build SES variable in studies related to health and SES (55-60). We studied the impact of family’s SES on child development and employed PFA model to create an index of family SES and create an overall SES variable from combination of related variables. 

The family SES index was evaluated using the following variables: parent’s education, household ownership, floor area of the housing unit, family property such as having one or two cars, household monthly income, having a computer, number of family members. PFA variable was an overall score and considering the qualitative nature of some variables in PCA, polychoric correlation matrix was used and an SES variable was created based on factor loadings. Then this score was categorized into five levels as follows: very low, low, moderate, high and very high, Logistic regression model was used to evaluate its relationship as an independent variable, as well as with controlling main underlying risk factors, to predict odds ratio of developmental delay with two codes, 0 and 1, as the outcome variables.

The data were analyzed using SPSS ver. 16 (Chicago, IL, USA) and Stata ver. 13. Mann Whitney test and logistic regression models were employed at significance level of 0.05.

All research ethical considerations were taken into account. The Ethics Committee of the University of Welfare and Rehabilitation Sciences confirmed the study. The participants were completely informed of the procedures and their informed written consents were obtained during data collection.

## Results

Family SES: PFA model was used to create an overall SES index and variables mentioned above were entered into the model. The first component had greater proportion in explaining variances in SES so that it explained about 75% of the total variance (Eigenvalue of 3.36). Table 1 presents the eigenvector corresponding to this component for different SES variables. The variables of “no personal computer” and “no car” had higher weight in comparison with other variables in the first principal component. 

A general score was determined for SES according to predict proxy and this score was divided into five levels (very low to very high or 1 to 5, respectively) and based on percentiles 40, 20, 60, 80.

The prevalence of developmental delay of the sample was 16.2% and there was a significant difference between different family SES levels at P=0.003, using Mann-Whitney U-test such that a decrease in SES resulted in an increase in the prevalence of developmental disorders (Table 2). Table 3 presents the developmental status in five dimensions with various levels of SES. 

The Mann-Whitney U test showed that dimensions of Communication (0.0001), Gross Motor (0.014) and Problem - Solving (0.021) were significantly correlated with SES.

The relationship between prevalence of developmental delay and various socioeconomic levels was evaluated using univariate logistic regression models and Odds Ratio (OR) of various levels compared to the highest group. OR of developmental delay increased with lower 

**Table 1:** Eigenvectors Components (Regression Ranking Coefficients

**Variables**	**Eigenvectors** **for first element**	**The portion of the total** **variance**	**Eigen value**
**Maternal ’s education**	0.168772	0.7543	3.36408
**Paternal’ s education**	0.08268		
**Income**	0.12104		
**Infrastructure**	0.07021		
**residential land price(M2)**	0.13745		
**no personal computer**	-0.28647		
**No car 1**	-0.29761		
**No car 2**	-0.8786		
**Number of family members**	-0.5682		
**Hosing**	-0.3338		

**Table 2:** Frequency of Developmental Delay in Children Aged 36-60 Months

**Domains**	**Normal Development** **Frequency(percent)**	**Delay Development** **Frequency(percent)**	**Total**
**LEVEL1**	155(74.5 )	53(25.5 )	208 (100.0 )
**LEVEL2**	176(85.0 )	31(15.0 )	207 (100.0 )
**LEVEL3**	181(87.4 )	26(12.6 )	207(100.0 )
**LEVEL4**	179(86.1 )	29 (13.9 )	208(100.0 )
**LEVEL5**	177(85.9 )	29(14.1 )	206(100.0 )
**TOTAL**	868(83.8 )	168(16.2 )	1036(100.0 )

Result of Mann-Whitney U* P*=0.003

**Table 3:** Relation of Socio -Economic Status and Developmental Delay with 5 Domains of Development

**Domains**	**Communication**		**Fine Motor**		**Gross Motor**		**Problem-** **Solving**		**Personal** **social**	
	**Delay** **(+)**	**Delay** **(-)**	**Delay** **(+)**	**Delay** **(-)**	**Delay** **(+)**	**Delay** **(-)**	**Delay** **(+)**	**Delay** **(-)**	**Dela** **(+)**	**Delay** **(-)**
**LEVEL1**	**27** **(13.0%)**	**181 (87.0%)**	**15** **( 7.2% )**	**193** **(92.8** **%)**	**18** **(8.7% )**	**190** **( 91.3% )**	**18** **(8/7%)**	**190** **(91.3%)**	**14** **(6.7%)**	**194** **(93.3%)**
**LEVEL2**	**21** **(10.1%)**	**186** **(89.9%)**	**8** **( 3.9% )**	**199** **( 96.1% )**	**10** **( 4.8% )**	**197** **(95.2%)**	**7** **( 3.4% )**	**200** **( 96.6%)**	**5** **(2.4%)**	**202** **(97.6%)**
**LEVEL3**	**7** **(3.4%)**	**200** **(96.6%)**	**8** **( ** **3.9%** ** )**	**199** **(96.1** **%)**	**11** **(5.3% )**	**196** **(94.7% )**	**7** **( 3.4% )**	**200** **( 96.6% )**	**7** **(3.4%)**	**200** **(96.6%)**
**LEVEL4**	**9** **(4.3% )**	**199** **(95.7%)**	**14** **(** **6.7%** ** )**	**194** **( ** **93.3%** ** )**	**9** **(4.3% )**	**199** **(95.7%)**	**9** **( 4.3% )**	**199** **(95.7% )**	**4** **(1.9%)**	**204** **(98.1%)**
**LEVEL5**	**8** **(3.9%)**	**198** **(96.1%)**	**13** **( 6.3% )**	**193** **(92.8** **%)**	**6** **(2.9% )**	**200** **(97.1% )**	**6** **(2.9% )**	**200** **( 97.1% )**	**7** **(3.4%)**	**199** **(96.6%)**
**TOTAL**	**72** **(6.9%)**	**964** **(93.1%)**	**58** **( 5.6% )**	**978** **( 94.4% )**	**54** **(5.2% )**	**982** **( 94.8% )**	**47** **( 4.5%)**	**989** **( 95.5% )**	**34** **(3.6%)**	**999** **(96.4%)**
**Result of** **Man-Witney** **test**	**0.0001**		**0.836**		**0.014**		**0.021**		**0.073**	

**Table 4:** Univariate Logistic Regression models, Relationship between SES Variables and Developmental Delay

**Socio- Eco** **Level**	**OR**	**P Value**	**CI**	
			**Lower**	**Upper**
**Level 1**	1	0.003		
**Level 2**	0.479	0.004	0.290	0.791
**Level 3**	0.930	0.796	0.538	1.608
**Level 4**	1.141	0.650	0.646	2.014
**Level 5**	1.011	0.968	0.581	1.762

**Table 5:** Multiple Logistic Regression Models, Relationship between SES Variables and Developmental Delay after Adjusting for the Following Factors: Child’s Age and Gender; Mother’s Gravida, Parity and Abortion

**Socio- Eco** **Level**	**OR**	** P Value **	**CI**	
			**Lower**	**Upper**
**Level 1**		0.0001		
**Level 2**	0.405	0.001	0.241	0.680
**Level 3**	0.786	0.408	0.455	1.390
**Level 4**	1.039	0.898	0.582	1.853
**Level 5**	1.024	0.935	0.584	1.795
**Girl** **Sex of child** **boy**	0.518	0.0001	0.367	0.730
**Age of child**	1.007	0.494	0.987	1.028
**Number of Gravida**	0.772	0.916	0.506	1.658
**Number of Abortion**	1.085	0.792	0.591	1.993
**Number of Parity**	1.332	0.373	0.708	2/505

SES. The distribution of developmental delay based on different SES levels and OR and 95% confidence intervals are shown in the Table 4.

A multiple logistic regression models were used to study the relationship between SES variables and developmental delay after adjusting for the following factors: child’s age and gender; mother’s gravida, parity and abortion. By referencing the first SES level and significance of the second level of this variable, the OR of developmental delay versus normal child development is 60% lower in the second level compared to the first. The child’s gender had a significant impact on their development and the OR of developmental delay in girls was 50% lower than in boys. (P=0.029) (Table 5).

## Discussion

The degree of developmental delay in children was 16.2%. Consistent with the study results; a prevalence rate of 16.3% was reported for developmental delay (32). A prevalence rate of 18% for developmental delay was reported in 4- to 60-month-old children from Tehran (30). This slight difference in reported prevalence rates can be attributed to different sample sizes and children’s age.

Family SES affected child development and a low SES was an independent risk factor for developmental delay. 

A decrease in family SES significantly increased the OR of developmental delay. Results of some studies are in line with our results (4, 20, 50, 52). However, most of the studies on the effect of SES on the developmental status of children have ignored the role of the main risk factors (32, 69, 70, 72). Many of these studies have only considered the role of income, education, and career,and have ignored the role of other factors.

Children of lower SES aged 36-60 months of age were more susceptible to developmental delay in ASQ Screening Test. SES mostly affected child development in communication, gross motor and problem solving domains.

Fine motor skills were the most delayed skills, and the least delayed skills were in the personal-social domain (41). This discrepancy in the results may be due to children’s age and different sample sizes.

SES in univariate and multivariate models showed a significant correlation only between developmental delay and child’s gender after controlling other factors such as child’s age, and mother’s gravida, parity and abortions. Children grown in families with lower SES, have their cognitive and emotional development and psychological health at higher risk (61).

Low family SES negatively affected child development particularly their communicational development (62).

The present study also found the highest developmental delay in communication skills. The impact of SES were reported on cognitive and emotional development in children (63).

SES is associated with a wide range of health outcomes, and cognitive and socio emotional development outcomes (3). The short-term (18, 19) and long-term (17, 22, 63, 64) were reported impacts of poverty on early childhood development. In addition, a strong relationship between risks factors of SES and long-term cognitive and linguistic outcomes rather than with socio emotional outcomes (65-67).

Low SES is associated with learning environment of children and it might affect their cognitive skills and developmental outcomes (5).

The relationship of low SES was reported at birth time with school achievements and cognitive skills of children (68). SES during infancy affected children’s cognitive development at 5 yr old (69).

Language deficit was reported in poor children aged 36-72 months in comparison with non-poor children. In addition, non-poor children achieved higher scores in vocabulary test than children with low SES (70). Great differences were reported in building vocabulary skills between children with high SES and low SES (71).

An experimental study on 3573 children aged 1 to 5 yr in Argentina showed that higher family SES and mother’s education were correlated with better psychomotor skills in children aged over 1 year (72).

Effective factors on developing gross motor skills in children are maternal education, socio economic status, physical environment (73-75). Moreover, there is no evidence in literature supporting the hypothesis that SES and children development (62). The positive impact of environmental stimulations in child development is well addressed in many studies in which secure attachment and a rich environment are considered as protective factors for child development (23, 76-79).

Brain development can be adjusted by the quality of environmental conditions (17) and brain can quickly develop through neurogenesis, axon/dendrite growth, synaptogenesis, apoptosis, synaptic pruning, myelination and glycogenesis (80).

Magnetic resonance imaging (MRI) has lately been used to show direct impact of SES on brain structure in children. Lower SES has been associated with smaller volumes of grey matter in some parts of brain. Brain structure can be affected by unfavorable environmental conditions even when deprivation and stress are not very severe (81).

Some scientists employed electrophysiological measures to study the impacts of SES on brain development.

This might show differences in cognition even when no differences in behavioral measures are observed.

In addition, baseline EEG activities have been used to evaluate general differences of brain function at resting state. EEG can be used to measure brain maturation especially in loci responsible for executive functioning (82). The differences in resting EEG patterns were reported in Mexican pre-school children because of SES function (83-84). On the other hand, SES affects brain development through different mediators such as parental factors, parental care, cognitive stimulations, nutrition, stress, etc. (85). Nutrition, home-environment, child-parent interaction, facilitating and stimulating learning experiences are effective on child development because higher SES would lead to better learning environment and lower SES is a barrier for learning and accessing cognitive stimulations (such as having access to newspapers, books, toys) (3).

Poverty would bring insufficient food and inadequate sanitation, which can lead to increased infection and stunting in children. It would also cause lower levels of education, an increase in depression and stress level in mother (3, 86, 87) and provide inadequate stimulation at home (88). A different approach was used and explained unfavorable impacts of SES on development via parents’ decisions for dedicating resources such as money, time and energy (money that parents would spend on buying books and toys, and the time spent with their children for reading books, etc.). Such measures are parents’ investment for child development and provide required potentials for cognitive and linguistic skills of children (89).

Studies have also indicated an increase in cortisol levels (90, 91). Chronic high levels of cortisol affect nervous system and other systems (92). There is a region in brain involved in stress response like prefrontal cortex, damaged at high levels of cortisol (93-96). Children with lower SES experience were documented higher levels of environmental and psychological stressors (3). 

On the other hand, parents with low SES have an increased risk of developing different psychological distress such as negative feelings of self-worth and depression symptoms (97). Depression can affect mother-child interaction and reduce developmental outcomes in social and cognitive domains (98, 99). Lower sense of responsibility in mothers is also associated with low income. As a result, factors that might increase mother’s stress level can act as a mediator in lower SES families (100). Moreover, children born in poor families are more exposed to unfavorable developmental conditions (such as living in crowded areas or slums and having bad neighbors) (101).

The positive impacts of environmental stimulations in relation to child development have been proved in other studies. Experiments on animals and children have shown secure attachment and rich environment are protective factors for child development. Moreover, some scientists have emphasized the existence of a relationship between SES and cognitive and linguistic learning of children through family simulations, number of siblings and number of family members who live together (62).

Under-five children who stay at home were more susceptible to developmental delays (62) though these impacts might be due to having less interaction and receiving less attention of parents, which in turn can reduce receiving environmental stimulations (3, 22, 23, 63, 102).

Babies who are surrounded with more people in the family have better gross motor skills because higher proportion of adult/children living together causes closer contact, hence, more stimulations are received associated with positive reactions in neuro psychomotor development (62).

One of the fundamental problems in evaluating SES and health is that data collection of qualitative variable such as income, family consumption or career is not easily possible thus, other proxies such as properties and housing are used (103,104).

Use of proxy measures can produce unreliable and unstable results because they show only a part of the overall impact. However, a combination of variables in PFA model helps quantify proxy variables, creates a measurable score for every individual, categorizes such quantitative scores, and determines SES level for each person (59, 105). Use of principal factor analysis is one of the strongest points of the present study. Therefore, fewer errors might occur in comparison with other methods based on weighting and people’s opinion. The current study used a combination of proxy variables to create an SES index, namely, family properties, residence price per square meter, the floor area of the housing unit, and the number of schooling years.

Findings of this study are limited to the mothers with kindergarten children.

**In conclusion, **it is necessary to implement interventions and preventive measures at family level to improve child development.

## Conflict of interest

This paper is adapted from a Ph.D. dissertation by Mahbobeh Ahmadi Doulabi entitled “Investigating the Status and Contribution of Parent Involvement in Children’s Education in Social Determinants of Health on the Developmental Level of 36-60 month-Old Children in Tehran.” The dissertation, no. 9958.1.T.93.801, has been written under the supervision of Dr. Firouzeh Sajedi and approved by the Pediatric Neuro Rehabilitation Research Center, University of Social Welfare & Rehabilitation Sciences. The authors declare that there is no conflict of interest. 
